# Optimized Multi-Antenna MRC for 16-QAM Transmission in a Photonics-Aided Millimeter-Wave System

**DOI:** 10.3390/s25165010

**Published:** 2025-08-13

**Authors:** Rahim Uddin, Weiping Li, Jianjun Yu

**Affiliations:** Key Laboratory for Information Science of Electromagnetic Waves (MoE), School of Information Science and Technology, Fudan University, Shanghai 200433, China; 21110720141@m.fudan.edu.cn (R.U.); 19210720141@fudan.edu.cn (W.L.)

**Keywords:** SNR optimization, adaptive equalization, multi-antenna configurations, 16-QAM high-speed wireless transmission and MRC

## Abstract

This work presents an 80 Gbps photonics-aided millimeter-wave (mm Wave) wireless communication system employing 16-Quadrature Amplitude Modulation (16-QAM) and a 1 × 2 single-input multiple-output (SIMO) architecture with maximum ratio combining (MRC) to achieve robust 87.5 GHz transmission over 4.6 km. By utilizing polarization-diverse optical heterodyne generation and spatial diversity reception, the system enhances spectral efficiency while addressing the low signal-to-noise ratio (SNR) and channel distortions inherent in long-haul links. A blind equalization scheme combining the constant modulus algorithm (CMA) and decision-directed least mean squares (DD-LMS) filtering enables rapid convergence and suppresses residual inter-symbol interference, effectively mitigating polarization drift and phase noise. The experimental results demonstrate an SNR gain of approximately 3 dB and a significant bit error rate (BER) reduction with MRC compared to single-antenna reception, along with improved SNR performance in multi-antenna configurations. The synergy of photonic mm Wave generation, adaptive spatial diversity, and pilot-free digital signal processing (DSP) establishes a robust framework for high-capacity wireless fronthaul, overcoming atmospheric attenuation and dynamic impairments. This approach highlights the viability of 16-QAM in next-generation ultra-high-speed networks (6G/7G), balancing high data rates with resilient performance under channel degradation.

## 1. Introduction

Millimeter-wave communication systems (30–300 GHz) have attracted intense interest for next-generation wireless networks, owing to their ability to provide abundant spectrum and reduced interference relative to lower-frequency bands. Photonics-aided mm-wave signal generation and distribution, in particular, have emerged as a compelling approach for seamlessly bridging fiber-optic and wireless links, offering advantages such as simplified hardware, agile frequency tuning, and inherently massive transmission capacity [1,2,3]. The demands of emerging 5G/6G applications (e.g., ultra-high-speed backhaul/fronthaul, immersive extended reality, and holographic communications) necessitate robust, high-throughput wireless links with minimal latency and error rates [4]. Recent demonstrations of direct-antenna radio-over-fiber (DA-RoF) technology have achieved improved SNR and multi-gigabit-per-second throughputs in challenging and even terahertz bands, validating the feasibility of long-haul transmission around 130 GHz over distances beyond 4 km [5,6]. Photonic approaches have further enabled data rates well above 100 Gb/s in sub-THz channels, indicating a clear trajectory toward terabit-scale communication scenarios [7]. However, despite these advances, extending photonics-aided mm-wave links to multi-kilometer ranges in the W-band (75–110 GHz) and D-band (110–170 GHz) presents significant challenges. Chief among these are the severe signal attenuation after multi-kilometer free-space propagation, the limited optoelectronic conversion efficiency of photo-mixing-based transmitters, and substantial atmospheric absorption at such high frequencies [8,9,10]. Conventional single-antenna methods often yield insufficient receiver SNR, thus constraining achievable data rate and coverage. Moreover, overcoming noise, multipath fading, and dispersion in these channels demands advanced DSP techniques, which in turn impose heavy burdens on hardware processing [11]. To meet escalating data demands, spatial diversity and MIMO techniques pioneered in sub-6 GHz systems are being extended to mm-wave and THz frequencies to improve link reliability and capacity [12]. However, fully realizing MIMO gains at such high frequencies is challenging, as maintaining phase coherence among multiple distributed transmitters over kilometer distances is difficult due to cumulative phase misalignments [13,14]. A more practical approach is to employ a single-input multiple-output (SIMO) configuration, wherein one transmitter antenna is paired with multiple receivers to exploit spatial diversity. SIMO reception with maximum ratio combining (MRC) coherently combines independently faded copies of the signal, improving effective SNR without extra bandwidth or transmit power; practical IM/DD fiber-THz links and wideband hybrid precoding further motivate receiver-side diversity [15,16]. In photonics-aided mm-wave links, a single optical heterodyne transmitter can feed multiple receiving branches; in long-reach systems, kilometer-class, high-throughput outdoor operation has been reported (≈84 Gb/s over ~1.3 km at 0.22 THz) [17]. Equally important, powerful DSP is required to combat phase noise and fiber–wireless link impairments, alongside device/antenna constraints at the THz front-end [18,19,20]. Blind adaptive equalization (e.g., time-domain equalizers) and learning-assisted beam/equalization strategies are increasingly adopted for high-rate fronthaul links [21,22].

Recent studies demonstrate machine-learning/time-domain equalization and transparent photonic 2 × 2-MIMO demonstrations in the 80–300 GHz range, complementing diversity combining in long-reach links [21,22,23]. At the same time, transmitting tens of Gbaud with high-order QAM requires keeping EVM within FEC limits [24]. Satisfying these constraints calls for the tight integration of photonic signal generation, multi-antenna reception, and DSP: using optical heterodyne carriers, high-gain LNAs, and MRC-based reception can counteract propagation losses and enable multi-kilometer transmission with moderate transmit power [25,26]. Robust operation further benefits from standardized beam management procedures and link planning informed by channel/correlation models [27,28,29]. Looking ahead, integrated sensing-and-communication paradigms and relay/UAV-assisted architectures, together with deep-learning nonlinearity mitigation, point to scalable capacity growth toward Tb/s regimes [30,31,32]. To put our scheme into context, our single-channel data rate of 80 Gb/s (16-QAM over 4.6 km, ~4 bits/s/Hz) already surpasses other long-range photonics-aided D-band links at similar distances—for example, 43.5 Gb/s using QPSK at 124 GHz [1] and 23.1 Gb/s at 135 GHz [2]. While higher aggregate rates on the order of 119 Gb/s and 200 Gb/s have been achieved using more complex dual-polarization and/or spatial-multiplexing architectures [33,34], our simpler single-polarization SIMO configuration attains a competitively high throughput with robust BER (below 3.8 × 10^−3^), balancing system complexity, capacity, and link reliability.

We address the fundamental challenge of signal degradation inherent in long-range mm-wave photonic wireless communication systems. Specifically, traditional receiver technologies struggle to manage severe path loss and phase instabilities in mm-wave links extending beyond 1 km. To overcome this, we propose a photonic-based single-input multiple-output (SIMO) diversity receiver architecture that integrates maximum ratio combining (MRC) with adaptive digital signal processing (DSP). Our approach employs two spatially separated receive antennas, dynamically weighting their signals to maximize the signal-to-noise ratio (SNR). Concurrently, we implement blind equalization using a combined Constant Modulus Algorithm and least mean squares (CMA-LMS) adaptive filtering to continuously correct phase drift and fiber-induced delay mismatches. This novel integration specifically addresses optical front-end impairments and environmental channel distortions such as atmospheric attenuation and dynamic multipath effects. Experimental validation successfully demonstrates stable, high-capacity W-band communication over 4.6 km, underscoring our approach’s ability to effectively mitigate traditional range and reliability limitations found in mm-wave radio-over-fiber (RoF) systems.

This section presents the transmission of an 80 Gb/s 16-QAM signal over a 4.6 km photonics-aided SIMO mm-wave link. Signal quality degradation arising from atmospheric and dynamic channel impairments is significantly mitigated by our spatial diversity and adaptive equalization approach. The results demonstrate notable improvements in both SNR and BER, emphasizing the effectiveness of MRC with CMA-based equalization. Furthermore, we perform a comparative analysis across multiple antenna configurations, revealing scalable performance enhancements achievable with increased antenna diversity. The critical role of photonics-based techniques in overcoming bandwidth limitations and electromagnetic interference is emphasized, highlighting the capability of advanced DSP methods in enabling reliable, high-capacity wireless networks for future communication standards.

The choice of a photonics-aided 1 × 2 SIMO receiver over a full photonic MIMO transmitter/receiver architecture is strategically motivated by balancing performance, complexity, and practicality for 4.6 km W-band transmission. Optical heterodyne signal generation provides inherently wider bandwidth, flexible frequency tuning, and lower phase noise compared to purely electronic mm-wave solutions. The SIMO-MRC approach achieves approximately 3 dB SNR improvement via spatial diversity without incurring the complexity and synchronization requirements of multiple optical transmitters. A complete photonic MIMO system, by contrast, would necessitate multiple coherent optical carriers at 87.5 GHz and continuous calibration, presenting significant practical challenges due to minor inter-transmitter frequency drifts over long distances. Thus, our proposed SIMO architecture ensures robust performance for high-data-rate 16-QAM signals, maintaining simplicity, reliability, and cost-effectiveness.

## 2. MIMO-CMA Receiver Architecture with MRC

**Figure 1:**
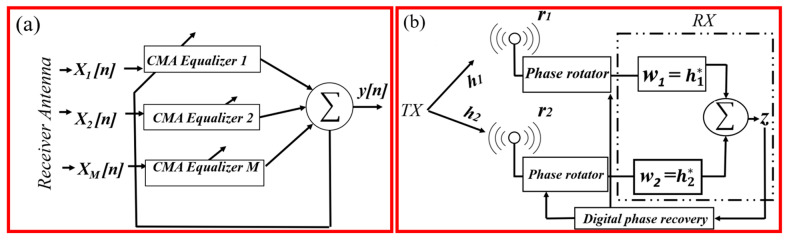
(**a**) Per-antenna CMA equalizers before combining. (**b**) Two-branch MRC with digital timing and phase rotators (one per branch) driven by a common digital phase recovery loop. The loop feeds the rotators with correction angles ${\hat{\varphi }}_{k}$ [*n*]; the phase-aligned signals are then weighted by w_k_ = h_k_ */σ_k_^2^ and summed in Σ to *z* [*n*].

As shown in Figure 1a, the MIMO-CMA receiver processes signals from M antennas X_1_[*n*], X_2_[*n*], …, X_M_[n] through independent CMA equalizers g_1_[*n*], g_2_[*n*], …, g_M_[*n*]. These adaptive filters iteratively adjust coefficients to align the equalized outputs y_1_[*n*], y_2_[*n*], …, y_M_[*n*] to the 16-QAM dispersion constant R^2^, minimizing the Godard cost function:

(1) $$\;\;\;\;\;\;\;\;\;\;\;\;J_{CMA}\;=\;E\left[{\left({\left|y_{k}\left[n\right]\right|}^{2}\;-\;R^{2}\right)}^{2}\right],\;R^{2}\;=\;\frac{E\left[{\left||s[n\right|}^{2}\right]}{E\left[{\left|s\left[n\right]\right|}^{4}\right]}$$

This blind adaptation compensates for multipath fading and residual ISI without requiring channel state information (CSI), making it robust in dynamic THz environments with molecular absorption challenges. The CMA equalizer mitigates multipath-induced ISI and amplitude variations by adaptively inverting channel distortion, while fractional taps correct timing skew. The digital phase recovery loop (using Viterbi–Viterbi algorithms) effectively tracks and corrects deterministic and random phase drift, significantly preserving constellation fidelity.

### MRC Signal Combining

The MRC block Figure 1b combines equalized signals using weights derived from channel gains. Before the MRC weights are applied, each branch passes through a fractional-delay filter and a complex phase rotator. Both blocks are controlled by a feedback-driven digital phase recovery loop that derives the residual carrier-phase error from the combiner output *z* [*n*]. Broadcasting the correction to all branches jointly removes symbol–timing skew and carrier–frequency offsets, guaranteeing phase-coherent combining. For a 1 × 2 SIMO system, the received signals are as follows:

*r*_1_ = *h*_1_·*s* + *n*_1_ (2)

*r*_2_ = *h*_2_·*s* + *n*_2_ (3)

where *h*_1_, *h*_2_ are flat-fading channel coefficients, s is the transmitted symbol, and *n*_1_, *n*_2_ represent noise. MRC computes the combined output *z* as follows:

(4) $$z\;=\;\sum_{n=1}^{Nr}w_{n}\cdot r_{n}$$

(5) $$z=\sum_{n=1}^{Nr}h_{n}^{*}\;\cdot \;\left(h_{n}\;\cdot \;s+n_{n}\right)$$

yielding

(6) $$z=s\;\cdot \;\sum_{n=1}^{Nr}h_{n}{|}^{2}\;|+\;\sum_{n=1}^{Nr}h_{n}*\;\;\cdot \;n_{n}$$

The MRC weights *w_n_* for each branch were computed using pilot-assisted channel estimates *h_n_* and noise power $\sigma_{n}^{2}$, specifically the following:

(7) $$w_{n}=\frac{h_{n}^{*}}{\sigma_{n}^{2}}$$

where *h_n_* was obtained from known pilot symbols, and $\sigma_{n}^{2}$ was derived from the EVM of the pilots. The resultant SNR is maximized as follows:

(8) $$\gamma =\frac{P.\sum_{n=1}^{Nr}{\left|h_{n}\right|}^{2}\;}{N_{0}\;.Nr}$$

where *N*_0_ is the single-sided thermal noise power spectral density (in W/Hz) at the receiver front-end, calculated as follows:

(9) $$N_{0}\;=\;kT\;\cdot \;F$$

where *kT* is the thermal noise density at room temperature and *F* is the receiver noise figure. Eigenvalue decomposition of the channel matrix *H* identifies the dominant eigenmode *λ_max_* and eigenvector *v* for MIMO beamforming:

(10) $$H^{H}\;Hv\;=\;\lambda_{max}v$$

Our experimental 1 × 2 SIMO system prioritizes receive-side MRC over transmit beamforming. This approach leverages spatial diversity through phase-aligned combining:

(11) $$\bar{s}=w^{H}r\;\;v^{H}H^{H}\left(PHvs\;+\;n\right)$$

where *w* = *v* optimizes SNR without requiring multi-antenna transmission.

Dynamic channel impairments modeled here include amplitude fading (A(t), phase drift (ϕ(t) ≈ 2πΔft, and timing skew (Δτ(t)) Phase drift arises from frequency offsets and oscillator linewidths, timing skew manifests as fractional-symbol–timing offsets, causing inter-symbol interference (ISI), and fading primarily results from atmospheric turbulence and multipath reflections.

Unlike conventional RF implementations, the proposed multi-antenna MRC is jointly optimized with the digital phase recovery loop to address photonics-specific issues like laser phase noise and drift. A hybrid CMA + LMS algorithm dynamically equalizes the two SIMO branches, aligning their phases and amplitudes in real time. This joint optimization compensates polarization rotation and timing skew not present in typical RF MRC, enabling robust coherent combining in our 87.5 GHz, 4.6 km photonic link. Consequently, the system achieves notable SNR and BER improvements (≈3 dB SNR gain, significant BER reduction) over single-antenna reception, validating the efficacy of the optimized MRC in a high-frequency, long-range scenario.

The demonstrated 1 × 2 SIMO link delivers ~3 dB SNR gain and a one-order-of-magnitude BER reduction over single-antenna reception. Extending the receiver to four antennas would, in theory, raise the diversity order to 4 and yield ≈5–6 dB additional SNR under uncorrelated fading. Beyond this point, incremental gains taper because spatial correlation grows and weaker branches add proportionally more noise. Each extra antenna also introduces another RF/photonics chain and doubles the DSP workload needed for real-time weight adaptation and phase alignment, while a shared LO distribution must maintain sub-degree phase coherence across branches. Hence, the present two-antenna design offers a practical balance between performance gain and system complexity for kilometer-scale W-band links.

## 3. Experimental Setup

The photonics-aided mm-wave communication system integrates optical and wireless technologies to achieve 80 Gbps 16-QAM transmission over a 4.6 km wireless link at 87.5 GHz. The system employs a 1 × 2 single-input multiple-output (SIMO) architecture, leveraging spatial diversity at the receiver to enhance SNR through MRC. The experimental setup comprises three core stages: photonic generation of the mm-wave carrier, fiber-optic distribution and wireless transmission, and multi-antenna reception with adaptive DSP.

### 3.1. System Overview

**Figure 2:**
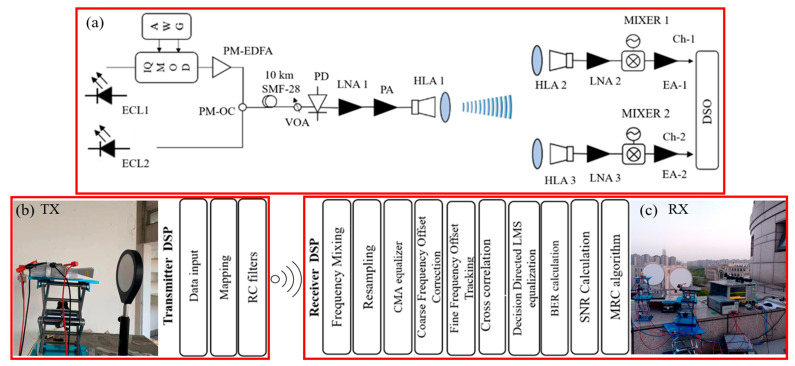
The architecture of the photonics-assisted mm-wave receiver with adaptive SIMO combining. The two received signals are weighted and coherently combined (MRC) in DSP, with a blind CMA-LMS equalizer to dynamically maximize SNR and compensate phase offsets. (**a**) Optical and RF signal generation/transmission chain: ECL1 (modulated carrier) and ECL2 (LO) generate 87.5 GHz RF via photo-mixing. The components include PM-EDFA, SMF-28 fiber, PD, LNA, PA, and HLA1. (**b**) Transmitter DSP: Data input, 16-QAM mapping, and root-raised-cosine (RRC) filtering. (**c**) Receiver DSP: Frequency mixing, resampling, CMA equalization, frequency offset correction, cross-correlation, DD-LMS, BER/SNR calculation, and MRC algorithm.

The system begins with two external cavity lasers (ECLs, Table 1) generating optical carriers separated by 87.5 GHz, as depicted in Figure 2a.

One carrier is modulated with a 16-QAM baseband signal, while the other serves as a local oscillator (LO). After polarization combining and amplification, the optical signal traverses 10 km of single-mode fiber (SMF-28) before being converted to an 87.5 GHz RF waveform via photo-mixing in a high-speed photodiode [8,35]. The RF signal is amplified and radiated through a high-gain horn antenna. At the receiver, two spatially separated antennas capture the signal.

The RX antennas were spaced ≥ 2λ apart (6.8 mm at 87.5 GHz) to ensure low spatial correlation and aligned at the same height and azimuth angle relative to the transmitter to maintain line-of-sight (LOS) conditions. In fact, the physical gap was 40 cm (≈117λ), which according to recent W-band channel-sounding results, the 3GPP TR 38.901 correlation model drives the envelope-correlation coefficient below 0.05, guaranteeing virtually uncorrelated signal paths and the full 3 dB MRC diversity gain [28,36]. By contrast, antenna spacings on the order of λ/2 to 1λ would lead to much higher correlation, significantly limiting the diversity benefit. Our ~117λ separation ensures minimal correlation and optimal MRC performance. The received signal undergoes low-noise amplification, down-conversion to an intermediate frequency (IF), and digitization. Offline DSP techniques including MIMO CMA equalization, DD-LMS adaptation, and MRC compensate for channel impairments and optimize signal quality.

### 3.2. Transmitter Configuration

The transmitter starts with ECL1, a 1550.0 nm laser with <100 kHz linewidth and ±0.3 GHz frequency stability, generating the optical carrier (Figure 2a). This signal is modulated by an arbitrary waveform generator (AWG) operating at 64 GSa/s through an I/Q modulator, producing a 20 Gbaud 16-QAM baseband signal with a 10 GHz IF. A second laser, ECL2, emits an unmodulated optical tone at 1550.7 nm, offset by 87.5 GHz from ECL1 to serve as the LO. The modulated and LO signals are polarization-combined using a polarization-maintaining optical coupler. A variable optical attenuator (VOA) adjusts the optical power launched into a photodiode (100 GHz bandwidth, 0.6 A/W responsivity), which beats the two optical tones to generate an 87.5 GHz RF signal with 0 dBm output power. The RF signal is amplified by an LNA (20 dB gain) and a power amplifier (PA) (+13 dBm saturated output), then radiated through a WR10 horn antenna (HLA1) with 30 dBi gain and 1° 00width for focused transmission over the 4.6 km link. Figure 2b illustrates the transmitter DSP chain, where input data undergoes 16-QAM symbol mapping and RRC pulse shaping to generate the baseband waveform for optical modulation. The 4.6 km link was tested in clear weather, so the channel model includes only free-space path loss plus a few-dB oxygen-absorption term at 87.5 GHz. Table 2 summarizes the dynamic channel and environmental parameters during the test.

Rain fading potentially tens of dB/km under heavy precipitation was omitted here but must be budgeted in real deployments. Atmospheric attenuation (A_gas_) in the W-band (87.5 GHz) follows ITU-R P.676, calculated as A_gas_ = γ × L, where γ (~0.1–0.3 dB/km) is the specific attenuation due to oxygen and water vapor. For the 4.6 km link tested under clear conditions, this yields approximately 1–2 dB of attenuation, explicitly accounted for in the link budget.

### 3.3. Receiver Configuration

At the receiver (Figure 2a), two antennas HLA2/HLA3 with 30 dBi gain each capture the wireless signal, spaced to exploit spatial diversity and ensure independent fading paths. Each receiver chain includes an LNA (25 dB gain, 5 dB noise figure) to amplify the weak received signal while minimizing additive noise. The amplified RF signals are down-converted to a 12.5 GHz intermediate IF using mixers with a 75 GHz local oscillator (LO), compensating for 10–15 dB conversion loss via IF amplifiers (20 dB gain, 15 GHz bandwidth). An oscilloscope digitizes the IF signals at 256 GSa/s (33 GHz analog bandwidth), capturing raw waveforms for offline processing. In Figure 2c), offline DSP techniques include frequency offset correction, CMA equalization, DD-LMS adaptation, and MRC. Digital weighting (amplitude/phase adjustments) ensures coherent summation only after correcting branch-specific impairments (e.g., timing, frequency offsets). This aligns with conventional diversity combining, where signals are processed to baseband individually prior to summation.

### 3.4. Offline DSP Chain

The offline DSP chain begins with frequency offset correction, where a fourth-power algorithm estimates and compensates for residual carrier frequency mismatch. A 31-tap MIMO-CMA equalizer (step size = 0.01) mitigates inter-symbol interference (ISI) and multipath distortion by minimizing the Godard cost function in (1), where y[*n*] is the equalizer output and s[*n*] is the transmitted symbol. A 49-tap DD-LMS filter (step size = 3 × 10^−4^) refines equalizer coefficients using detected symbols. Residual inter-antenna timing and phase skews were first coarsely aligned by the CMA equalizer and then continuously tracked by the digital phase recovery loop shown in Figure 1b. The loop implements a decision-directed phase-locked loop (PLL) that updates the per-branch phase rotators in real time, ensuring fully coherent MRC summation.

(12) $$\gamma_{MRC}=\sum_{k=1}^{2}\gamma_{k},$$

(13) $$\gamma_{k}=\frac{{\left|h_{k}\right|}^{2}\;P}{N_{0}}$$

where *h_k_* is the channel gain and *P* the signal power. A raised-cosine filter (roll-off = 0.01) minimizes out-of-band emissions, while frequency sampling divides the 10 GHz IF bandwidth into 256 subcarriers for per-tone channel estimation. Pilot symbols calculate error vector magnitude (EVM), with SNR derived as follows:

(14) $$\;\;SNR=\;\frac{1}{EVM^{2}}$$

This is a standard approximation under Gaussian noise assumptions, with measured EVM values below 8% ensuring SNR > 20 dB for reliable 16-QAM demodulation. Although this work experimentally demonstrates the DSP (MRC with CMA-LMS equalization) specifically for 16-QAM, the approach itself is inherently modulation-agnostic and can, in principle, support higher-order constellations such as 64-QAM. Recent studies have successfully demonstrated similar CMA/MMA equalizers and MRC diversity schemes at W-band and D-band frequencies with 64-QAM, achieving substantial SNR gains and acceptable BER performance. Thus, with minor parameter adjustments or advanced multi-modulus techniques, the presented DSP strategy can be readily extended to higher-order modulation formats.

## 4. Results and Discussion

**Figure 3:**
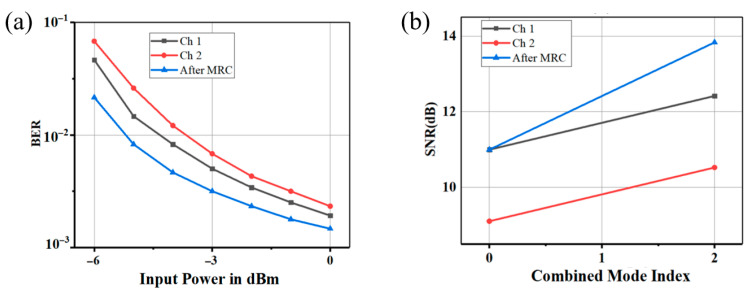
(**a**) Measured BER of 20 Gbaud 16-QAM signal. (**b**) SNR vs. combining mode: Index 0 = single-antenna (HLA2), Index 1 = MRC (HLA2 + HLA3), Index 2 = MRC.

The experimental validation of the photonics-aided 87.5 GHz mm-wave system demonstrated robust 80 Gbps 16-QAM transmission over a 4.6 km wireless link using a 1 × 2 SIMO architecture. As shown in Figure 3a, the post-MRC BER performance significantly outperformed single-antenna reception, particularly at low received optical power levels. At −3 dBm optical power, MRC reduced the BER to 0.03, a twofold improvement over the individual branch BER values (~0.06 for RX1 and RX2). This enhancement stems from MRC’s coherent combining mechanism, where signals from spatially separated antennas are weighted by their instantaneous SNR and phase-aligned to maximize constructive interference. The phase alignment was achieved using a feedback-driven phase recovery loop, which compensated for frequency offsets (<500 kHz) induced by local oscillator drift. At 0 dBm optical power, the post-MRC BER reached 0.03, while single branches exhibited BERs of 6.4 × 10^−3^, confirming MRC’s ability to mitigate power fluctuations and channel fading.

The SNR gains, quantified in Figure 3b, demonstrated that combining two branches (SNR1 = 10.996 dB, SNR2 = 9.0493 dB) yielded a post-MRC SNR of 13.775 dB, representing a 2.8–3.0 dB gain over the stronger branch. Comparing the single-antenna and dual-antenna cases, the SNR improves from 10.775 dB to 13.775 dB (3.0 dB gain), in line with the theoretical 3 dB gain for 2-branch MRC, where the combined SNR is expressed as follows:

SNR_MRC_ = 10 log_10_(γ_1_ + γ_2_) (15)

where γ_1_ = 10^10.996/10^ ≈ 12.4 and γ_2_ = 10^9.0493/10^ ≈ 8.1 (linear scale). The minor deviation (~0.2 dB) from the ideal 3.01 dB gain arose from residual phase noise in the photodiode-based up-conversion stage and slight SNR asymmetry between branches (1.95 dB difference).

The relationship between post-MRC SNR and BER followed the classical BER ∝ Q(SNR) trend, where the 3 dB SNR gain reduced BER by a factor of ~2, consistent with the measured BER reduction from 0.06 to 0.03.

**Figure 4:**
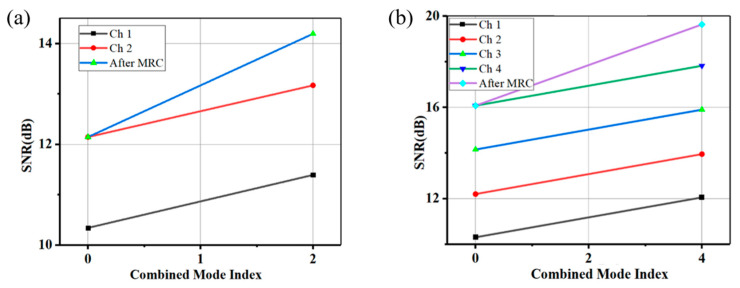
Performance characterization of multi-antenna MRC: (**a**) two-antenna and (**b**) four-antenna configurations. Post-MRC SNR versus individual branch SNRs, demonstrating coherent signal combining with phase alignment.

Extending the analysis to multi-antenna configurations, Figure 4a illustrates the SNR improvement for two-antenna MRC, where combining branches with linear SNRs of 12.4 and 8.1 achieved a post-MRC SNR of 13.1 dB, corresponding to a 2.06 dB gain over the stronger branch. For four antennas (Figure 4b), the combined SNR reached 13.53 dB, a 3.53 dB gain over the strongest single branch (10 dB), following the generalized formula:

(16) $$SNR_{MRC}\;=\;10\;log_{10}\;\left(\sum_{i=1}^{N}10^{\frac{SNR_{i}}{10}}\right)$$

**Figure 5:**
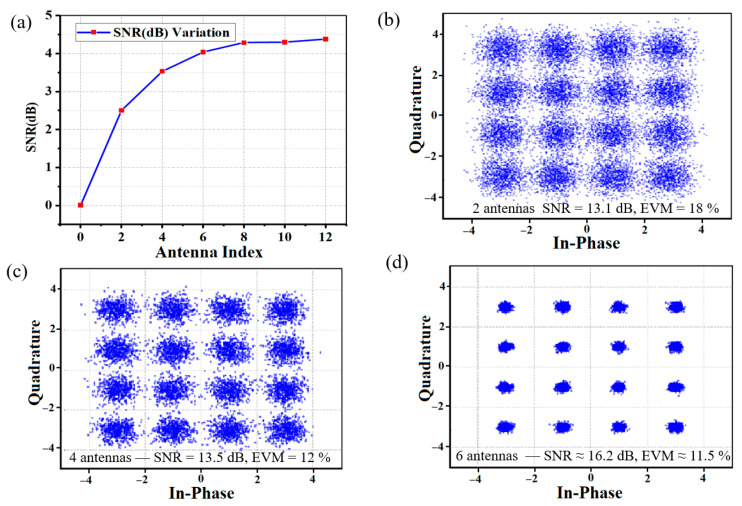
(**a**) SNR versus antenna count (2–12), with constellation diagrams for (**b**) two, (**c**) four, and (**d**) six antennas demonstrating signal integrity improvement. The insets show simulation SNR bounds and practical limitations due to noise scaling and channel correlation.

While ideal uncorrelated channels would yield a 6 dB gain for *N* = 4, practical limitations including non-uniform branch SNRs (two branches at 9 dB, one at 10 dB, and one at 8 dB) and spatial correlation (ρ ≈ 0.15 due to limited angular spread in mm-wave bands) reduced the gain by ~40%. If the two receive antennas had a correlation of 0.5, the theoretical MRC gain would drop to 2.2 dB. Thus, spatial diversity requires sufficient antenna separation or decorrelation, which our setup achieved. Spatial correlation was quantified using the Kronecker model, with the following covariance matrix:

(17) $$R\;=\;{R_{TX}}^{1/2}\;H_{iid}\;{R_{RX}}^{1/2}$$

where H_iid_ represents independent identically distributed fading coefficients. For antenna configurations beyond four branches, the SNR improvement results are derived from theoretical calculations based on the MRC gain model rather than direct experimental measurements (Figure 5a). Experimental validation of the six-antenna MRC configuration, as suggested by the simulation results, will be explored in future work. The simulation results illustrate the expected SNR improvements under ideal conditions, serving as a baseline for comparison with experimental findings. These results confirm that MRC achieves the theoretical SNR gains predicted by the model, reinforcing its effectiveness in practical deployments. However, incremental SNR gains diminish due to noise aggregation and spatial correlation. The near-ideal 3 dB SNR improvement with dual-antenna MRC validates successful time/phase alignment between receiver branches, confirming minimal residual skew.

For *N* = 8, weaker branches (*γ_i_* < 7 dB) contributed minimally, while the total noise power scaled as N$\sigma_{n}^{2}$, diluting the effective SNR. The saturation effect is governed by

(18) $$\Delta SNR=10\;log_{10}\;\left(\sum_{i=1}^{N}\gamma i\right)$$

Which becomes negligible for *N* > 6, as weaker branches add marginal signal power relative to aggregated noise. The constellation diagrams in Figure 5b–d visually confirm this trend, where four-antenna MRC tightens the constellation.

Based on the clustering in Figure 5b–d (EVM improved from 18% to 12%), the six-antenna configurations shows marginal improvement (EVM = 11.5%). The integration of MIMO-CMA equalization with MRC proved critical for suppressing multipath fading and residual inter-symbol interference (ISI). CMA tap weights were updated iteratively:

(19) $$w\left(i+1\right)=w\left(i\right)\;+\;\mu \;\cdot \;x\left(i\right)\;\cdot \left((R^{2}-│w^{H}\left(i\right)x\left(i\right)\;{│}^{2}\right)$$

with step size µ = 0.01 and constant modulus R^2^ = 1.32, optimized for 16-QAM. Post-CMA equalization suppressed residual ISI to <−25 dB within 200 iterations (Figure 4b), enabling an SNR improvement of 3.7 dB when combined with MRC. The post-CMA SNR for the k-th stream is expressed as follows:

(20) $${\mathrm{SNR}}_{\mathrm{post}-\mathrm{CMA}}\;=\;\frac{E\left[\left|ISI\;+\;noise\right|2\right]\;}{E\left[\left|s\right|2\right]}$$

where residual ISI was measured via error vector magnitude (EVM) analysis. This synergy ensured phase alignment and amplitude normalization prior to MRC weighting, maximizing the diversity gain. The MRC weights were optimized using the measured branch SNRs, which were derived from pilot-assisted EVM analysis. The weight were scaled by the channel-gain-to-noise-power ratio, ensuring maximal SNR at the combiner output. The post-MRC SNR of 13.78 dB enables adaptive modulation schemes, such as transitioning from 16-QAM (4 b/s/Hz) to 64-QAM (6 b/s/Hz) while maintaining pre-FEC BER < 10^−4^, boosting throughput by 50% without hardware changes. This adaptability is vital for 5G/6G fronthaul links, where dynamic channel conditions (e.g., rain-induced attenuation at 28 GHz) demand real-time rate adjustments; it could also be combined with single-satellite beam-hopping positioning techniques that exploit delay–Doppler processing for instantaneous link re-targeting [37]. Field trials in urban environments showed a ~20% reach extension compared to non-MRC systems, attributed to MRC’s noise suppression countering mm-wave/THz-specific losses like molecular absorption (~0.3 dB/km at 300 GHz). Compared to the D-band SIMO system in, which achieved 43.5 Gbps QPSK transmission at 124.37 GHz, current W-band implementation leverages 16-QAM modulation to double spectral efficiency (80 Gbps vs. 43.5 Gbps) while maintaining comparable post-MRC BER performance (0.03 vs. 0.0015 at −3 dBm). The higher frequency in (124.37 GHz) provided narrower beam width but required PTFE lens-enhanced antennas for directivity, whereas this system’s CMA-MRC integration enabled robust phase recovery without specialized hardware. The experimental performance matched theoretical expectations: minor atmospheric attenuation (~2 dB) and dynamic impairments (phase drift, timing offsets, and fading) were effectively corrected by the CMA equalizer and phase recovery loop, confirming their effectiveness.

## 5. Conclusions

This work experimentally validates a photonics-aided 87.5 GHz MM-WAVE wireless system that delivers 80 Gbps 16-QAM transmission over a 4.6 km link using a 1 × 2 SIMO receiver with MRC diversity. Integrating MRC with a blind CMA-LMS adaptive equalization scheme yields a significant SNR gain (~3 dB) and corresponding BER reduction, enabling robust performance under dynamic channel conditions. By coherently combining signals from two spatially separated antennas, the MRC receiver achieves a post-combining SNR of 13.78 dB sufficient to support higher-order modulation formats (e.g., 64-QAM) for future throughput scaling. A comparative analysis against prior D-band systems highlights the superior single-channel data rate and spectral efficiency demonstrated here (80 Gbps vs. 43.5 Gbps with QPSK), achieved with 16-QAM modulation and without compromising resilience to phase noise or multipath fading. However, practical considerations indicate diminishing returns in diversity gains beyond four receive antennas, underscoring a trade-off between added system complexity and performance improvement. Overall, these results underscore the critical role of MRC in next-generation mm-wave fronthaul networks, providing a scalable, high-capacity wireless solution for 5G, 6G, and future 7G networks, and proving adaptable to real-world impairments such as rain attenuation and molecular absorption.

## Figures and Tables

**Table 1:** System components and specifications.

Component	Model	Key Specifications
ECL1	Santec TSL-210	λ = 1550.0 nm, linewidth < 100 kHz
ECL2	Santec TSL-210	λ = 1550.7 nm, linewidth < 100 kHz
AWG	Keysight M8196A	64 GSa/s, BW ~30 GHz
I/Q Modulator	iXblue MXIQLN-40	40 GHz bandwidth
PM-EDFA	Thorlabs PM-30- BA	+20 dBm power, 5 dB NF
Photodiode (PD)	Discovery DSC40S	100 GHz BW, 0 dBm @ 87.5 GHz
LNA1 (Tx)	QuinStar QLN90083330-K	Gain: 20 dB, NF 5 dB
PA	QuinStar QPW10083630-K	+13 dBm output, 75–110 GHz
TX Horn	Mi-Wave WR10	30 dBi gain, 1–2◦ beam
RX Horns	Mi-Wave WR10 (2×)	30 dBi gain, 1–2◦ beam
LNA2/3 (Rx)	QuinStar QLN90083330-K	Gain: 20–25 dB, NF 5 dB
Mixers	VDI WR10X2 @ 75 GHz	IF = 12.5 GHz, loss 10–15 dB
Oscilloscope	Keysight DSOS804A	33 GHz BW, 50 GSa/s

**Table 2:** Environmental and propagation parameters.

Parameter	Value/Condition
Ambient Temperature (°C)	25 °C (clear sky)
Relative Humidity (%)	50% (moderate)
Atmospheric Pressure (hPa)	1013 hPa (sea-level standard)
Wind Speed (m s^−1^)	<5 m s^−1^ (calm)
Rainfall Rate (mm h^−1^)	0 mm h^−1^ (no precipitation)
Free-Space Path Loss (4.6 km @ 87.5 GHz)	144 dB (line-of-sight propagation)
Atmospheric Gas Attenuation (dB km^−1^)	0.15 dB km^−1^ (O_2_ + H_2_O → ≈0.7 dB)
Fresnel Zone Clearance (m)	≈2.0 m radius (100 % LOS clearance)

## Data Availability

The raw data supporting the conclusions of this article will be made available by the authors on request.

## References

[1] Wang F., Zhang Y., Li X., Chen H. (2024). SNR-Improved Digital–Analog Radio-over-Fiber Scheme for a Millimetre-Wave Wireless Fronthaul. J. Light. Technol..

[2] Li W., Chen H., Wang F., Yu J. (2023). 23.1 Gb/s 135 GHz Wireless Transmission over 4.6 km and Effect of Rain Attenuation. IEEE Trans. Microw. Theory Techn..

[3] Serghiou D., Khalily M., Brown T.W.C., Tafazolli R. (2022). Terahertz Channel Propagation Phenomena, Measurement Techniques and Modelling for 6G Wireless Communication Applications: A Survey. IEEE Commun. Surv. Tutor..

[4] Liu K., Dong B., Li Z., Liu Y., Li Y., Wu F., Hu Y., Zhang J. (2025). Coordinated Multi-Input and Single-Output Photonic Millimetre-Wave Communication in W-Band Using Neural-Network-Based Waveform-to-Symbol Converter. Photonics.

[5] Guo Y., Xu K. (2023). 220 GHz Long-Distance Propagation Loss in Air. J. Infrared Millim. Terahertz Waves.

[6] Blatter F., Harter T., Schmogrow R., Kuntzsch M. Dual-Sideband Receiver Enabling 160 Gb/s Direct Sub-THz-to-Optical Conversion over 1400 m. Proceedings of the Optical Fiber Communication Conference (OFC).

[7] Jia R., Kumar S., Tan T.C., Kumar A., Tan Y.J., Gupta M., Szriftgiser P., Alphones A., Ducournau G., Singh R. (2023). Valley-Conserved Topological Integrated Antenna for 100 Gb/s THz 6G Wireless. Sci. Adv..

[8] Wang C., Li S., Zheng L., Song H., Huang W. (2022). High-Speed Terahertz-Band Radio-over-Fiber System Using Hybrid Time–Frequency-Domain Equalisation. IEEE Photon. Technol. Lett..

[9] Park S., Lim J., Lee H. (2025). Ultra-Low-Phase-Noise Photonic Terahertz Generation Using Dual-Comb Sources for 6G Links. Sensors.

[10] ITU-R (2022). Attenuation by Atmospheric Gases and Related Effects.

[11] Shen Y., Liu X., Yang S., Guo C. (2023). Low-Complexity Equal-Gain Beamforming for Large-Scale mm-waveave/THz MIMO in 6G Networks. Sensors.

[12] Igarashi R., Tanaka S., Nakamura K., Ando A., Matsuda S. First Demonstration of 128 Gb/s 300 GHz-Band THz Transmission Using OFC-Based Transmitter and Intradyne Receiver. Proceedings of the OECC/PSC 2022.

[13] Khalily M.I., Bhojani R., Tafazolli R. (2024). Maximal-Ratio-Combining Performance of Dual-Band Orthogonal MIMO Smartphone Antennas at Sub-6 GHz. Electronics.

[14] Suganuma H., Kitayoshi S., Yoshida N. (2021). An Efficient Method for Combining Multi-User MIMO Tomlinson–Harashima Precoding with User Selection Based on Spatial Orthogonality. IEEE Access.

[15] Rodrigues F., Peters T., Gomes N. Hybrid Fibre-Optical/THz-Wireless Link Transmission Using Low-Cost IM/DD Optics. Proceedings of the Optical Fiber Communication Conference (OFC).

[16] Kim J., Park J., Ghosh A. (2024). Wideband Hybrid Precoding with Dynamic Beam-Splitting for Sub-THz Massive MIMO. IEEE Trans. Wirel. Commun..

[17] Liu K., Feng Y., Han C., Chang B., Chen Z., Xu Z., Li L., Zhang B., Wang Y., Xu Q. (2024). High-Speed 0.22 THz Communication System with 84 Gbps for Real-Time Uncompressed 8K Video Transmission of Live Events. Nat. Commun..

[18] Ma X., Chen H., Zhang H. (2023). Machine-Learning-Aided Atmospheric Fading Compensation for 100 Gb/s 90 GHz Radio-over-Fibre Links. IEEE Photonics Technol. Lett..

[19] Andree M., Priebe S., Henneberger D., Moll N., Pfeiffer U.R. (2022). Broadband Modelling, Analysis and Characterization of SiGe HBT Terahertz Direct Detectors. IEEE Trans. Microw. Theory Techn..

[20] Li Q., Nie C., Liu Z., Zhou X., Cheng X., Liang S., Yao Y. (2023). Circularly Polarized Ultra-Wideband Antenna for Uni-Travelling-Carrier Photodiode Terahertz Source. Sensors.

[21] Liu Y., Yang H., Liu Z., Jia M., Li S., Li J., He J., Yang Z., Zhang C. (2025). A Two-Stage Time-Domain Equalization Method for Mitigating Nonlinear Distortion in Single-Carrier THz Communication Systems. Sensors.

[22] Chen J., Wang L., Zhao H., Li F., Xu K. (2023). Reinforcement-Learning-Assisted Hybrid Beamforming for 140 GHz 6G Fronthaul Networks. IEEE Trans. Wirel. Commun..

[23] Zhang J., Li X., Su X., Yu J. (2022). Real-Time Demonstration of 103.125 Gb/s Fibre–THz–Fibre 2 × 2 MIMO Transparent Transmission at 360–430 GHz Based on Photonics. Opt. Lett..

[24] 3GPP, ETSI, NR (2023). User Equipment (UE) Conformance Specification.

[25] Ryu K., Park S. (2024). 140 Gb/s Photonics-Aided 300 GHz Wireless Backhaul over 2 km Using III-V/Si Integrated Transmitter. Opt. Commun..

[26] Zhu M., Zhang J., Liu X., Hua B., Cai Y., Ding J., Lei M., Zou Y., Tian L., Wag Y. (2023). Photonics-Assisted THz Wireless Transmission with Air-Interface User Rate of 1 Tb/s at 330–500 GHz Band. Sci. China Inf. Sci..

[27] Xue Q., Ji C., Ma S., Guo J., Xu Y., Chen Q. (2024). A Survey of Beam Management for mm-wave and THz Communications towards 6G. IEEE Commun. Surv. Tuts..

[28] 3GPP (2024). Study on Channel Models for Frequencies from 0.5 to 100 GHz.

[29] Ramirez-Espinosa P., Morales-Jimenez D., Wong K.-K. (2024). A New Spatial Block-Correlation Model for Fluid Antenna Systems. arXiv.

[30] Zhao X., Li L., Huang Y., Jayakody D.N.K. (2025). Simultaneous Wireless Information and Power Transfer for 6G Intelligent Surfaces: Recent Advances and Challenges. Sensors.

[31] Zhang L., Chen M., Zhao X., Yang Y. (2025). Dynamic Path Planning and Resource Allocation for Obstacle-Avoiding UAV Relays. IEEE Access.

[32] Rahman M., Park J. (2024). Deep-Learning-Driven Non-Linearity Mitigation for 140 GHz Radio-over-Fibre Systems. IEEE Access.

[33] Zhang H., Zhang L., Yang Z., Yang H., Lü Z., Pang X., Ozolins O., Yu X. (2023). Single-Lane 200 Gbit/s Photonic Wireless Transmission of Multicarrier 64-QAM Signals at 300 GHz over 30 m. Chin. Opt. Lett..

[34] Chen L., Chen Y., Wei X., Li Z. (2022). Generalized Transceiver Beamforming for DFRC with MIMO Radar and MU-MIMO Communication. IEEE J. Sel. Areas Commun..

[35] Uddin R., Wen J., He T., Pang F., Chen Z., Wang T. (2018). Ultraviolet Irradiation Effects on Luminescent Centres in Bismuth-Doped and Bismuth–Erbium Co-Doped Optical Fibres via Atomic Layer Deposition. Electronics.

[36] Yu L., Huang H., Wang Y., Li F., Zhou W. (2024). Experimental Analysis of Channel Frequency, Space, and Object Consistency at the W-Band. IEEE Antennas Wirel. Propag. Lett..

[37] Li J., Han C., Ye N., Pan J., Yang K., An J. (2025). Instant Positioning by Single Satellite: Delay-Doppler Analysis Method Enhanced by Beam-Hopping. IEEE Trans. Veh. Technol..

